# Styrene-Butadiene Rubber by Miniemulsion Polymerization Using In Situ Generated Surfactant

**DOI:** 10.3390/polym12071476

**Published:** 2020-06-30

**Authors:** Anderson M. S. Medeiros, Elodie Bourgeat-Lami, Timothy F. L. McKenna

**Affiliations:** Univ Lyon, University Claude Bernard Lyon 1, CPE Lyon, CNRS, UMR 5265, Chemistry, Catalysis, Polymers and Processes (C2P2), 43 Bvd. Du 11 Nov. 1918, F-69616 Villeurbanne, France; andersonmmsm@gmail.com (A.M.S.M.); bourgeat@lcpp.cpe.fr (E.B.-L.)

**Keywords:** SBR, latex, miniemulsion, gel content, high solids content, elastomer

## Abstract

An alternative approach for the synthesis of styrene butadiene rubber (SBR) copolymer latexes was explored in order to obtain low gel fractions and high solid contents. The ultra-turrax-assisted miniemulsion stabilized by in situ surfactant generation was adopted as the main strategy since this technique can inhibit the eventual presence of secondary nucleation producing polybutadiene particles and also control the cross-linking degree. Styrene monomer was first miniemulsified using an ultra-turrax and in situ generated surfactant using either hexadecane (HD) or octadecyl acrylate (ODA) as the hydrophobe. Dynamic light scattering (DLS) measurements of droplet size indicated faster stabilization and the production of smaller droplet diameters ca. 190 nm (PdI = 0.08) when employing in situ generated potassium oleate (K-Oleate) in comparison to SDS-based miniemulsions. High butadiene-level SBR latexes with ca. 50% solids content, a glass transition temperature (*T*_g_) of −52 °C, and a butadiene to styrene weight ratio of 75:25, were then obtained using the miniemulsion droplets as seeds. Turbiscan and DLS measurements revealed a very stable resulting latex with SBR particle diameter of ca. 220 nm and a low polydispersity index (PdI). Secondary nucleation was prevented as indicated by the low N_p_/N_d_ value. Cryo-TEM images showed a narrow distribution of particle size as well as the absence of agglomeration. The gel content was below 10% when tert-dodecyl mercaptan (t-DM) was used as chain transfer agent (CTA).

## 1. Introduction

Styrene butadiene rubber (SBR) is one of the most widely produced synthetic rubbers, and accounts for around 40% of the global synthetic elastomer production [1]. The styrene-to-butadiene ratio and its microstructure (1,4-*cis*, 1,4-*trans*, and 1,2-vinyl) play important roles in determining the final properties of SBR [2,3]. From a commercial point of view, high butadiene-level SBR latexes with high *cis*/*trans* ratios and low gel contents are targeted since both contribute to increase the SBR’s processability. 

The degree of cross-linking is certainly the most widely discussed of SBR properties, since high amounts of cross-linking can make it less processable and affect its viscoelastic properties [4,5,6]. The 1,2-vinyl addition is elected as the principal responsible for the increase of the level of branching and, consequently, the increase of cross-linking. Two main strategies are typically adopted to reduce or control the cross-linking degree: (1) using a chain transfer agent (CTA) and (2) stopping the reaction at around 70% conversion since from that point, the monomer-to-polymer ratio is low, encouraging cross-linking [4].

Classical emulsion polymerization (hot or cold process) is the most widely employed method to produce SBR latexes. The hot process takes place between 50–70 °C, and potassium persulfate (KPS) is commonly used as initiator. In the cold process (performed at 5 °C), a redox initiator is used, such as chelated iron/organic peroxide and sodium formaldehyde sulfoxide (SFS) [7]. The latter route produces a yellow-colored SBR as consequence of the presence of redox initiator. Both routes usually involve mainly sodium dodecyl sulfate (SDS) as a surfactant [6].

The synthesis of SBR latexes by miniemulsion polymerization has also been investigated [4,5]. In miniemulsion polymerization, each nanodroplet acts as a nanoreactor and particle nucleation takes place inside monomer droplets. In principle, homogenous nucleation of particles can be minimized by using a hydrophobic initiator [8]. When coupled with the use of a hydrophobe to retard the Ostwald ripening process it is possible to obtain stable miniemulsions. The use of in situ generated surfactant instead of a pre-formed one can also contribute to the reduction of secondary nucleation, as this strategy leads to less free surfactant since the major part will be formed on the surface of the particles when the base in the water phase neutralizes the fatty acid at the oil phase interface. Furthermore, compared to classical emulsion polymerization, miniemulsion allows the production of polymers with lower degrees of cross-linking (and consequently lower gel contents). At high conversions, the miniemulsion technique produces around 70 wt% of gel fraction whereas conventional emulsion polymerization results in 90 wt% gel content. This is due to the fact that in the earlier stages in miniemulsion, the high monomer-to-polymer ratio inside the nucleated miniemulsion droplets is expected to reduce the level of branching comparing to emulsion polymerization and delay the formation of gel to higher conversions [4].

Li et al. performed a comparative kinetic study of SBR in miniemulsion and conventional emulsion polymerization [4]. They showed that secondary nucleation of butadiene was prevented in miniemulsion polymerization, while in conventional emulsion polymerization, nucleation occurred both in micelles and homogeneously in the continuous phase. Similarly, Landfester et al. investigated the impact of temperature (from 30 °C to 72 °C) on the properties of SBR latexes synthesized by miniemulsion polymerization using different hydrophobic initiators [5]. The authors concluded that SBR latexes produced by miniemulsion exhibited low gel contents and the molar mass could be easily tuned by using a CTA. In addition, they noticed that the microstructure was not significantly affected by the temperature or the quantity of CTA.

A concern in miniemulsion polymerization is maintaining the intrinsic stability of the dispersion of droplets during the reaction. The miniemulsification process plays a key role in the achievement of stable nanodroplets. Typically, an ultrasound apparatus is used on a laboratory scale as shearing device because it is a simple and fast method to generate the nanodroplets. However, the homogenization by sonication is limited to small amounts of emulsion and low viscosities, as the majority of energy input during the shearing process of highly viscous mixtures is dissipated as excessive heat production (due to viscous resistance) [9]. As a consequence of these limitations, this method is not practical at commercial scales. On the other hand, the mechanisms behind the operation of an ultra-turrax provokes the circulation of fluid throughout the vessel ensuring the mixing cycle during the shearing process [10].

Associated with the miniemulsification process, the stability is achieved by using a surfactant in combination with a hydrophobe, the role of which is to retard Ostwald ripening [11,12,13]. A considerable amount of research has shown the importance of the surfactant and hydrophobe in miniemulsion polymerization. An appropriate hydrophobe should have a low molar mass (to maximize the hydrophobe-to-monomer molar ratio into the droplets), high monomer solubility (to maximize the hydrophobe-monomer interaction parameter), and low water solubility (to ensure high level of the hydrophobe into the droplets, limiting the rate of monomer diffusion out of the smaller droplets) [11]. Studies involving free-radical polymerization via miniemulsion have often used hexadecane (HD) as a hydrophobe [14,15,16,17]. As an alternative to HD, reactive hydrophobes such as stearyl methacrylate (SMA) as well as highly hydrophobic polymers have been used [16,17,18]. 

Bearing this discussion in mind, the current work focused on the synthesis of high butadiene-level SBR latexes with high solids and low gel content via miniemulsion polymerization using rotor-stator as high-shear mixer. The rotor-stator is more suitable for industrial production than ultrasound [19]. Furthermore, an in situ generated K-oleate surfactant was used as it could stabilize the newly produced droplets more quickly than conventional surfactants, and create stable miniemulsions with high solid contents, as reported by El-Jaby et al. [20]. The use of a reactive hydrophobe was also considered in order to reduce the levels of volatile organic compounds (VOC).

## 2. Experimental 

### 2.1. Chemicals and Reagents

The monomers: styrene (99%, Sigma-Aldrich, St. Quentin, Fallavier, France), butadiene (Air liquide), the initiator 2,2′-azobis(2-methyl butyronitrile) (VAZO 67, 98% Sigma-Aldrich, St. Quentin, Fallavier, France), hexadecane (HD, 98%, Fisher, Illkirch, Strasbourg, France), octadecyl acrylate (ODA, 97%, Sigma-Aldrich, St. Quentin, Fallavier, France), oleic acid (90%, Sigma-Aldrich, St. Quentin, Fallavier, France), potassium hydroxide (KOH, 85%, Acros Organic, Illkirch, Strasbourg, France), and tert-dodecylmercaptan (t-DM, 98.5%, t-DM, Sigma-Aldrich, St. Quentin, Fallavier, France) were all used as received. Deionized water (DDI) was used in all polymerization experiments.

### 2.2. Preparation of SBR Latex by Miniemulsion Polymerization 

**Figure 1:**
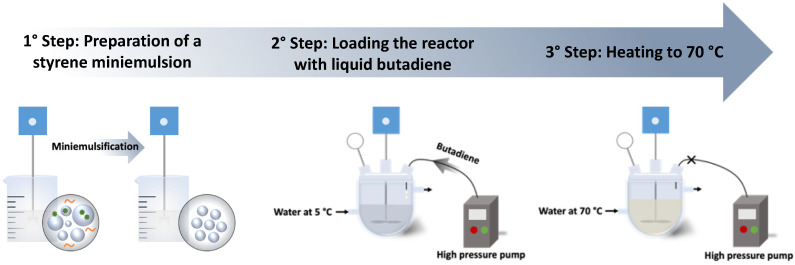
Scheme of production of styrene butadiene rubber (SBR) latexes by seeded miniemulsion polymerization.

First of all, a pre-formed styrene miniemulsion (recipe described in Table 1) was added to a 450 mL Parr reactor and cooled to 5 °C. The system was kept under stirring (100 rpm) for fifteen minutes to reach thermal equilibrium. Then, liquid butadiene (65 g or 102 mL) (density of butadiene is given in Table 2) was added via a high-pressure pump and kept for thirty minutes for homogenization under mechanical stirring (200 rpm). Finally, the reactor was heated to 70 °C ± 2, the agitation was increased to 350 rpm and the polymerization conducted overnight. The increase of temperature to 70 °C was accompanied by the increase of the pressure to around 8 bar. The scheme in Figure 1 illustrates the general experimental procedure.

In order to determine the solids content of the latex, gravimetric analyses of each sample were performed three times. Approximately 1 g of the latex was weighed in a previously weighed aluminum pan and kept in an oven at 100 °C for 24 h.

### 2.3. Characterizations 

The SBR samples were named HD-SBR, ODA-SBR, NCo-SBR for SBR synthesized using HD, ODA, and in absence of the hydrophobe, respectively. As the recipes were subjected to reproducibility reactions, #01, #02, and #03 were attributed to samples made in triplicate.

#### 2.3.1. Dynamic Light Scattering (DLS)

Hydrodynamic diameters (D_h_) were determined by dynamic light scattering (DLS) in a Malvern Zeta-Sizer Nano-ZS. Note that this apparatus gives an average of the intensity of the diameters present in the sample. Samples were diluted in DDI water prior to the analysis. For each sample, three measurements of twelve runs each were performed at 25 °C to obtain the D_h_ and the size dispersity (PdI). The PdI is a value provided by the Malvern instrument and it is used to describe the width of the particle size distribution around a central value. A value of less than 0.1 indicates a very narrow size distribution.

#### 2.3.2. Cryo-Transmission Electronic Microscopic (Cryo-TEM)

The particle size and morphology were determined by cryogenic transmission electron microscopy (cryo-TEM) using a Philips CM120 transmission electron microscope from the Centre Technologique des Microstructures (CTμ), platform of the Université Claude Bernard Lyon 1, in Villeurbanne, France. For cryo-TEM analysis, a drop of the latex was deposited without dilution onto 300 mesh holey carbon films (Quantifoil R2/1) and quench-frozen into liquid ethane, using a cryo-plunge workstation (made at LPS Orsay). The specimens were then mounted on a precooled Gatan 626 specimen holder, and observations were made at an accelerating voltage of 120 kV. Statistical analyses of particle size were performed on approximately 500 particles from cryo-TEM micrographs. The number and weight average particle diameters (*D*_n_ and *D*_w_, respectively) and the polydispersity index (*D*_w_/*D*_n_) were calculated using *D*_n_ = Σn_i_*D*_i_/Σn_i_ and *D*_w_ = Σn_i_*D*_i_^4^/Σn_i_*D*_i_^3^, where n_i_ is the number of particles with diameter *D*_i_.

#### 2.3.3. Nuclear Magnetic Resonance (^1^H NMR and ^13^C NMR)

Samples were characterized by liquid ^1^H NMR and ^13^C NMR spectroscopy (300 MHz Bruker) in deuterated CDCl_3_ at room temperature. Both methods were used to identify the SBR composition as well as its microstructure in terms of 1,4-*trans*, 1,4-*cis*, and 1,2-vinyl addition.

#### 2.3.4. Differential Scanning Calorimetry (DSC)

The glass transition temperature (*T*_g_) of the copolymers was determined by DSC using a DSC 3 from Mettler Toledo. The analysis of approximately 10 mg of each sample was performed in the temperature range of −90 to 120 °C, with a heating rate of 10 °C min^−1^ and a N_2_ flow of 30 mL min^−1^.

#### 2.3.5. Size Exclusion Chromatography (SEC)

SEC measurements were conducted in THF at 40 °C with a flow rate of 1 mL min^−^. The separation was carried out on three columns from PSS Instruments (PSS SDV analytical (8 × 300 mm)). The device (Viscotek TDA305) was equipped with an RI detector (λ = 670 nm) and the average molar masses and molar mass distributions were derived from the RI signal using a polystyrene calibration curve. 

#### 2.3.6. Swelling Experiments

The gel content was determined by extraction with toluene at 50 °C for 24 h. In a typical experiment, approximately 100 mg of sample were weighed and dissolved in 20 mL of toluene. The mixture was stirred for 24 h at 50 °C, and filtered through a 100-μm-pore-size filter. Finally, the soluble fraction was determined by gravimetric analysis as follows:

(1) $$\mathrm{GC}=\frac{w_{d}}{w_{0}}\times 100$$

where *w*_0_ is the mass of the sample before swelling, and *w_d_* the mass of dried sample after filtration.

#### 2.3.7. Turbiscan^®^ Measurements

Measurements of sedimentation formation were conducted using the Turbiscan Lab^®^ (Formulaction). Approximately 15 g of each sample (latex) was kept in a specific glass flask and was analyzed using the scanning mode continuously for three days. Scans were recorded every seventeen minutes.

## 3. Results and Discussion

### 3.1. Stability of Styrene Miniemulsions

**Figure 2:**
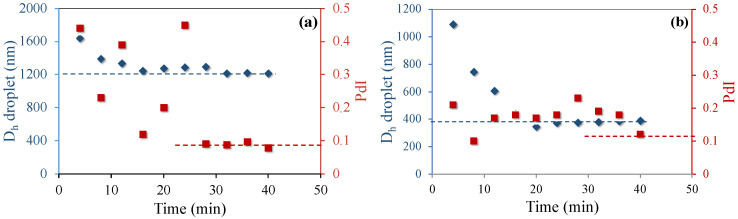
Progress of dynamic light scattering (DLS) droplet diameter over time for styrene miniemulsions obtained using the rotor–stator mixer operating at a rotational speed of 3000 rpm with different surfactants: (**a**) 1.5 wt% of SDS and (**b**) 1.5 wt% of in situ generated K-oleate, using HD as the hydrophobe.

**Figure 3:**
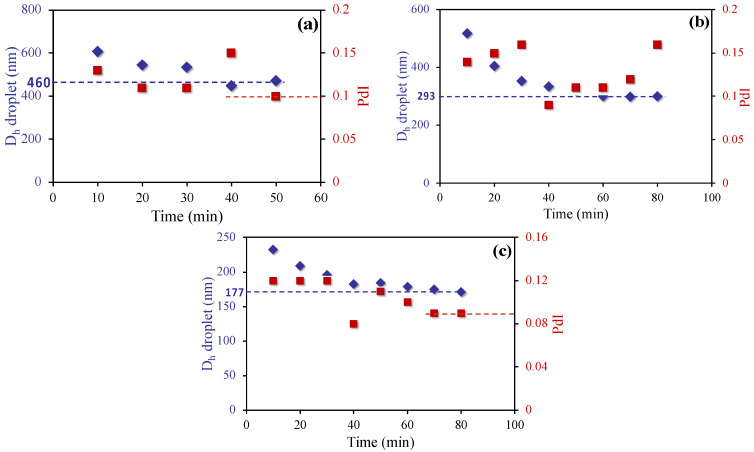
Evolution of droplet diameter as a function of time for styrene miniemulsions prepared using the ultra-turrax (rotational speed at 20,000 rpm) as emulsification device and hexadecane (HD) as the hydrophobe. (**a**) 1.5 wt.% of SDS (**b**) 3 wt% of SDS, and (**c**) 3 wt% of in situ generated K-oleate.

A key initial step to producing a stable SBR latex by miniemulsion polymerization is to be able to form stable styrene miniemulsion droplets. The styrene mixture indicated in Table 1 was miniemulsified by using 2 miniemulsification devices: (1) a VMI Rayneri TURBOTEST rotor stator mixer with a rotational speed range of 50 to 3300 rpm with a stator head equipped with 4 blades angled at 90° and measuring 5.5 cm in diameter; and (2) an ultra-turrax LABOMODERNE DHX 350D with a rotational speed range between 4000 and 33,000 rpm equipped with a 30 mm-stator head model DHX 30C. Different surfactants were tested and the droplet diameters were monitored by DLS. The resulting DLS measurements were plotted over time as depicted in Figure 2 and Figure 3.

As seen in Figure 2, in both cases, the droplets made with the rotor stator mixer reached a minimum size after approximately 20 min. The limiting size for the system with the in situ generated K-oleate was around 420 nm, meanwhile using pre-formed SDS, it was about 1200 nm. The energy provided by this device was not sufficient to produce droplets small enough to continue with SBR polymerization using SDS as surfactant. 

As shown in Figure 3, replacing the rotor-stator operating at 3000 rpm with ultra-turrax operating at 20,000 rpm, but keeping everything else the same, allowed us to reduce the droplet diameter from 1200 to 460 nm (compare Figure 2a and Figure 3a). Increasing the SDS level to 3 wt% led to a further reduction in the droplet size down to around 290 nm. In case of using 3 wt% of the in situ K-oleate, the droplet diameter was smaller than 200 nm. In addition to being available to promptly stabilize the newly produced droplets, the high stabilization capacity of in situ generated surfactant can also be attributed to the chain length of oleic acid (18C) compared to SDS (12C). These observations are in agreement with those of El Jaby et al. [20] confirming the ability of in situ generated K-oleate to create stable miniemulsions when associated to the ultra-turrax device.

In order to have further information about the stability of the styrene miniemulsion, the shelf-life was monitored by DLS as depicted in Table 3.

The results shown in Table 3 reveal that the coalescence and diffusional degradation were successfully inhibited with the use of in-situ generated K-oleate providing an efficient long-term stability to the styrene droplets. This behavior is valuable from a commercial point of view as it suggests that the miniemulsions can be prepared and stored for several days before use. Several studies have described the formation of miniemulsions using in situ generation surfactant [21,22,23]. In all cases, the authors state that they obtained stable miniemulsions, but did not investigate the long-term stability of the products.

### 3.2. Synthesis of High Solids Content SBR Latexes

The SBR latexes were synthesized after loading the pre-formed styrene miniemulsion with liquid butadiene following the recipe in Table 2. The polymerization reaction was carried-out overnight for total conversion. An SBR latex was first synthesized by using in situ generated K-oleate and HD as surfactant and hydrophobe, respectively (sample HD-SBR#01). The pH of the SBR latexes was around 11.2 due to the presence of KOH excess.

**Figure 4:**
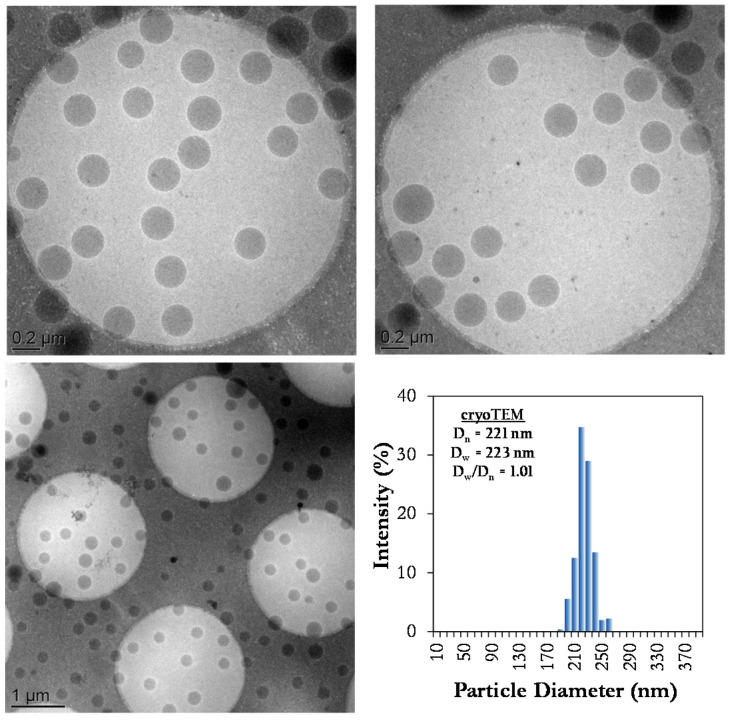
Cryo-TEM images and corresponding size distribution histogram of HD-SBR#01.

DLS was used to characterize the size and size dispersity of the SBR latex particles. The SBR latex had an average particle diameter of the order of 218 nm, and a PdI = 0.04, which can be considered very low (conventionally a PdI < 0.1 is taken to be indicative of a narrow particle size distribution). The cryo-TEM images of Figure 4 support this conclusion and show the formation of monodisperse particles with a diameter of ca. 220 nm, in agreement with DLS analysis, and a very narrow size distribution (*D*_w_/*D*_n_ = 1.01). There is no visual evidence of small particle typically associated with homogeneous nucleation, nor do the DLS analyses reveal any small particles (see Figure S3 in the Supplementary Materials). The increase of particle size when compared to the droplet diameter (from 177 nm to 218 nm) can, in large part, be attributed to the incorporation of the butadiene in the initial drops. In addition, considering that a nearly one to one copy of droplets to particles was obtained (N_p_/N_d_ = 1.3) (for equations see Supplementary Materials), it can be assumed that nucleation occurred preferentially in the droplets and that homogeneous nucleation was minimized or absent. 

An estimated overall conversion of 95% and a solids content of 46% were obtained by gravimetric analysis. The conversion was calculated by taking into account the experimental and theoretical solids content. It should be emphasized that butadiene is a gas at the reaction temperature and cannot thus considered in the solids content.

The composition and microstructure of the SBR latex were determined by ^1^H NMR and ^13^C NMR, as reported in the literature [24,25,26]. The results are shown in Table 4.

As expected, the resulting SBR latex exhibited high content of butadiene as shown in Table 4. Furthermore, the ^13^C NMR results indicated a rich *trans/cis* SBR microstructure. The low amount of 1,2-vinyl addition contributed to the low gel content of the SBR latex. The literature reports that a SBR with similar composition and microstructure shows a *T_g_* of around −50 °C [5].

By DSC measurements, a single glass transition temperature (*T_g_*) attributed to the SBR copolymer was identified at around −52 °C (Figure S1, Supplementary Materials). The presence of only one *T_g_* indicates the synthesis of a homogeneous statistical copolymer. In addition, taking into account the composition obtained by ^1^H NMR, the experimental *T_g_* was in agreement with the calculated *T_g_* estimated by the Fox equation [27] (*T_g_* (calculated) −48 °C) according to:

(2) $$\frac{1}{T_{g,mix}}=\overset{j}{\underset{i}{\sum }}\frac{\omega_{i}}{T_{g,i}}$$

where *ω_i_* is fraction of monomer *i*, and *T_g_*_,*i*_ is the glass transition temperature of the homopolymer produced from monomer *i*.

The slight difference between the experimental and calculated *T_g_* can be attributed to the *cis/trans* and vinyl ratio. Indeed, the Fox equation does not take into account the microstructure, and the *T_g_* can be influenced by this ratio, and it is at best a rough approximation of the real *T_g_*. In addition, the co-stabilizer can also act as a plasticizer leading to a decrease of the experimental *T_g_*. 

**Figure 5:**
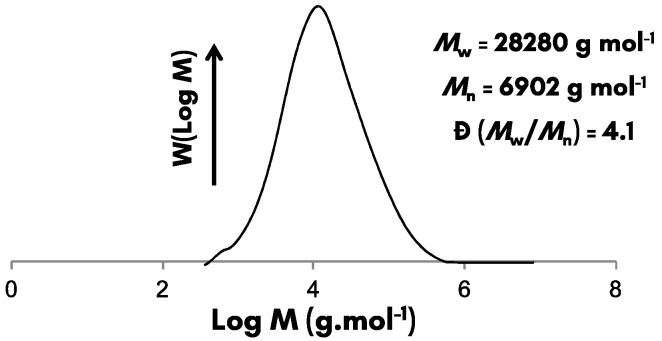
Molar mass distribution of the HD-SBR#01 with 4 wt.% of tert-dodecyl mercaptan (t-DM).

To complete the characterization of the resultant SBR latex, SEC measurements and swelling tests were performed. The SEC chromatogram of the soluble fraction of HD-SBR#01 using 4 wt% of t-DM as CTA is shown in Figure 5 while the results of the insoluble gel content (replicate polymerizations) are given in Table 5.

The molar mass of SBR was around 2.8 × 10 ^4^ g mol^−^ (Ð = 4.1) (Figure 5). The low resulting molar mass was likely the consequence of the high amount of CTA used to control the gel content, which induced the deactivation of macroradicals. Thiol-based compounds are indeed known to be very efficient chain transfer agents [28]. Another point that might have influenced the decrease of the molar mass was the significant amount of initiator (2 wt%, see Table 2) used in the process. SEC curves comparing the molar mass of SBRs synthesized with different amounts of CTAs are depicted in Figure S2 and Table S2. As expected, the chromatograms revealed a diminution of both the molar mass and Ð with increasing the CTA amount.

As shown in Table 5, the extractions resulted in an average of insoluble amounts of 5.5%. Considering the amount of t-DM employed in the reaction (4 wt%), this result is completely reasonable since, according to the literature, the CTA controls the molar mass of the polymer as well as the gel content [28]. As reported by Moustafa and co-authors, the cross-linking in SBR is mainly caused by 1,2-vinyl additions which are responsible for introducing reactive pendant double bonds which in turn can easily react with active radicals [17]. In addition, Charmot and Guillot observed a decrease of SBR gel content with increasing CTA amounts due to deactivation of macroradicals interrupting polymer chain growth [6]. The impact of the CTA is confirmed for runs with a concentration of 2 wt%, where the gel content was found to be around 22% as shown in Table S1 (Supplementary Materials).

### 3.3. Effect of the Nature of the Hydrophobe

As discussed previously, an SBR latex was initially synthesized using HD as the hydrophobe (see recipe in Table 2). The experiment was performed in triplicate and the results are summarized in Table 6. Three replicate polymerizations were run under the same conditions but using ODA as the hydrophobe. The results of these runs are compared to the HD runs in Table 6.

As observed in Table 6, the properties of HD-SBR were similar in all cases which suggests a very reproducible system. Since the miniemulsion technique allowed us to reproducibly make an SBR latex with the desired properties, the next challenge was to replace HD which is a volatile organic compound by octadecyl acrylate (ODA) (a reactive hydrophobe). 

**Figure 6:**
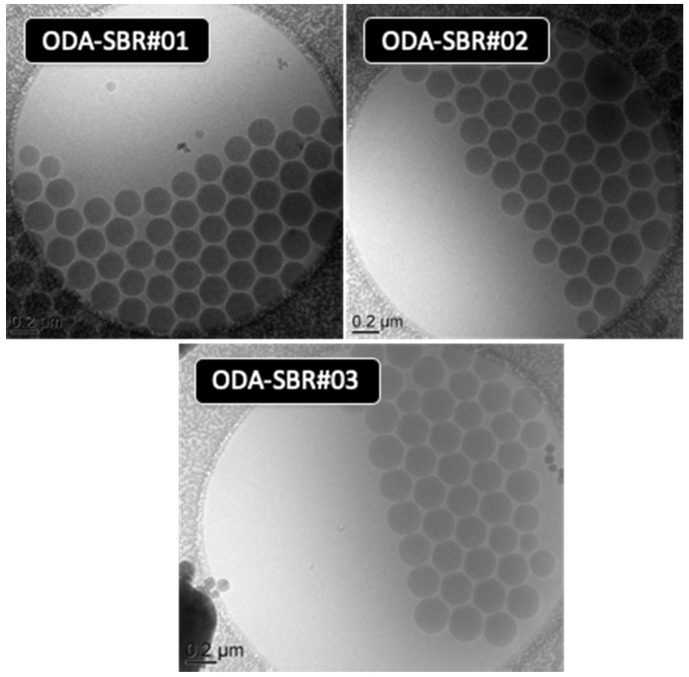
Cryo-TEM images of SBR latexes produced by miniemulsion using ODA as the hydrophobe.

In the case of ODA-SBR, the reactions were again very reproducible and presented similar *T*_g_, gel content, composition and microstructure as the ones using HD (see Table 6). The average particle size at the end of the polymerization was also similar for both sets of runs, where the average D_h_ of the particles made with HD was 218 nm (PdI = 0.03) and the average D_h_ of the particles made using ODA was 218 nm (PdI = 0.01). Both latexes had a low size dispersity as indicated by the very low PdI values, and confirmed by cryo-TEM (Figures S3 and S4, Figure 4 and Figure 6). Furthermore, the droplet diameter for ODA-SBR was around 168 nm with a PdI value of 0.015 (N_p_/N_d_ = 1.2) compared to HD-SBR (D_h_ droplets ~ 180 nm, PdI = 0.01). In addition, the weight average molar mass for the soluble ODA-SBR was about 4.7 × 10^4^ g mol^−1^, instead of 3 × 10^4^ g mol^−1^ for HD-SBR. It is possible that this difference is due to the slightly lower gel content for ODA-based polymers.

**Figure 7:**
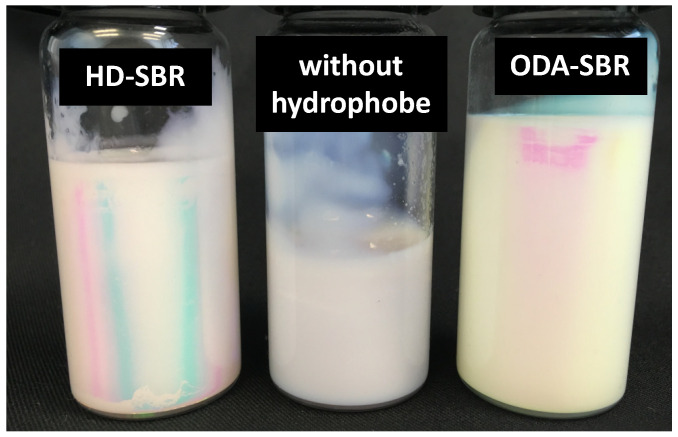
Macroscopic aspects of the SBR latexes synthesized based on the recipe in Table 2, using HD or ODA as the hydrophobe or without hydrophobe.

Another possibility explored was to test whether or not the CTA was an adequate hydrophobe since it is itself a very hydrophobic compound and was present in high concentration. However it was found that the polymerization was not reproducible enough and the latex showed macroscopic coagulation during the reaction. The instability was clearly evident in the cryo-TEM images (Figure S7) that showed the presence of big particles likely formed by diffusional degradation as a result of the absence of the hydrophobe. Thus, it appears that DM alone is not a suitable hydrophobe, at least for the current system. The macroscopic aspects of the SBR latexes obtained by using different hydrophobes are shown in Figure 7.

The samples are highly viscous due to the presence of high solids content and the low *T_g_* of the polymer. One sample prepared without co-stabilizer showed a presence of clots corroborating previous results, as evidenced in Figure 7. It is important to note that these samples showed viscosities compatible with a commercial SBR latex with similar properties (solids content, *T_g_*, and gel content).

**Figure 8:**
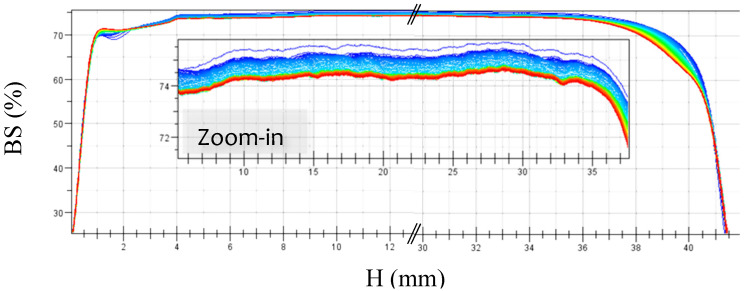
Turbiscan^®^ measurements of the HD-SBR#01 latex.

**Figure 9:**
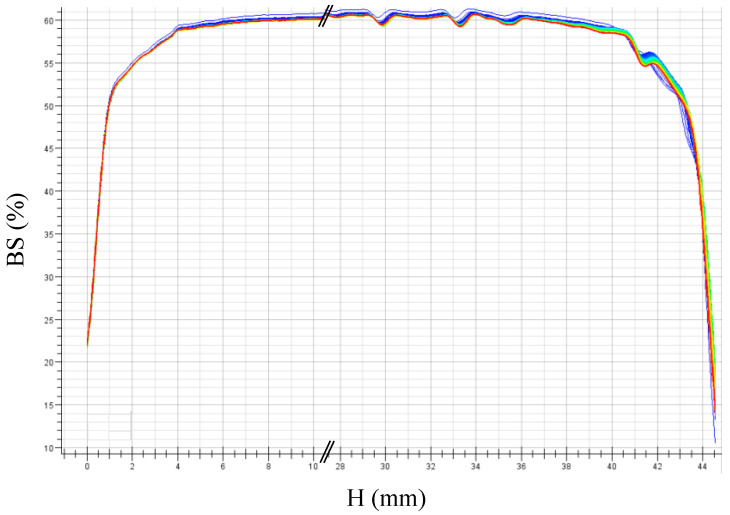
Turbiscan^®^ measurements of ODA-SBR#01 latex.

Finally, the stability and shelf life of the HD-SBR and ODA-SBR latexes were monitored by Turbiscan^®^ measurements over 2 days, and the results are displayed in Figure 8 and Figure 9.

The very slight shift in the backscattering signal over time (2 days) for both latexes ODA-SBR or HD-SBR shows that they are both stable for a period of days. There is no visible coalescence, nor measurable settling of the final latexes. 

### 3.4. Scaling Up of SBR Miniemulsion Polymerization

Scaling up experiments were run in order to evaluate possible large-scale production of SBR latexes through the polymerization of miniemulsions produced using the ultra-turrax as high-shear mixer. The properties as well as stability of up-scaled SBR latexes (total weight = 3 kg) were investigated and the data is shown in the Supplementary Materials (Figures S5 and S6). The scaling up reactions were performed using HD or ODA as the hydrophobes. Both hydrophobes resulted in stable SBR latexes with around 45% solids content and the same properties as those obtained on the lab scale.

## 4. Conclusions

The assisted-ultra-turrax miniemulsion stabilized by in situ generated surfactant used in this work successfully resulted in stable SBR latexes with high solids content and low crosslinking degree. The N_p_/N_d_ and the narrow particle size distribution indicated the efficient droplet nucleation as well as there is no evidence of fine particles being formed which suggest the minor presence or absence of homogeneous nucleation.

Stable SBR latexes with around 50% solids content, particle diameter of 220 nm, *T_g_* of −52 °C, high butadiene content (butadiene-to-styrene ratio = 75/25), and low insoluble gel fractions (*ca.* 4 wt%) were synthesized by miniemulsion polymerization. Thanks to in situ generated K-oleate, small droplet diameters could be obtained using ultra-turrax as shearing device enabling the production of SBR latex by miniemulsion on a commercial scale. DLS measurements revealed a droplet diameter of ca. 180 nm with low PdI. The miniemulsions were shown to be stable over a period of several days. The use of t-DM as CTA enabled to successfully control the insoluble gel amount and also the molar mass. The low gel amount provides good processability to SBR, which is very important to the industrial process. The estimated overall conversion based on solids content was 95%.

The replacement of HD by ODA as the hydrophobe also resulted in a stable SBR latex with similar properties. In the latter case, the molar mass increased due to the slightly lower gel content for ODA-based polymers.

Scaling up experiments were performed resulting in SBR latexes with similar properties as the ones produced on the lab scale, regardless of the nature of the hydrophobe (either HD or ODA). These results showed that it was possible to achieve stable SBR latexes in a commercial scale with desired properties and avoid using a VOC as the hydrophobe. The obtained SBR latexes were again very stable and the polymerization highly reproducible and, consequently, this strategy can be considered as an alternative approach to produce SBR latexes on a commercial scale.

## Figures and Tables

**Table 1:** Styrene miniemulsion formulation.

Chemicals	m (g)	wt% Based on Styrene
Styrene	30	-
Deionized water	100	-
Hexadecane or Octadecylacrylate	1.2	4
Oleic Acid	0.9	3
Potassium hydroxide ^a^	0.3	-
tert-Dodecylmercaptan	1.2	4

^a^ Oleic acid to potassium hydroxide molar ratio = 2:3 [20].

**Table 2:** Seeded SBR miniemulsion formulation.

Chemicals	Weight (g)	wt% Based on Organic Phase
Styrene	30	-
Butadiene ^a^	65	-
Deionized water	100	-
Hexadecane or Octadecylacrylate ^b^	1.2	1.2
tert-Dodecylmercaptan ^c^	3.8	4
VAZO 67	1.9	2
Oleic Acid ^b^	0.9	1
Potassium hydroxide ^d^	0.3	-

^a^ ρ (butadiene) at 5 °C = 0.64 g cm^−3^. ^b^ The amounts of the hydrophobe and oleic acid (based on styrene) were 4 wt% and, 3 wt%, respectively. ^c^ In some specific cases, 2 and 3 wt% of tert-Dodecylmercaptan were used to evaluate the gel content formation. ^d^ Oleic acid to potassium hydroxide molar ration = 2:3 [20].

**Table 3:** Evolution of the droplet diameter with time for a styrene miniemulsion prepared using the rotor-stator mixer and the recipe in Table 1 with HD as the hydrophobe.

Time (Days)	D_h_ (nm) ^a^	PdI ^a^
0	177	0.08
1	174	0.09
2	177	0.08
3	171	0.09
4	174	0.07
7	184	0.08

^a^ Determined by DLS.

**Table 4:** Results of SBR composition and microstructure of HD-SBR#01 obtained from ^1^H and ^13^C NMR.

Poly(butadiene-*co*-styrene)			
Butadiene (%)			Styrene (%)
87.2			12.8
1,4-*cis*/1,4-*trans* (%)		1,2-vinyl (%)	
71.4		15.8	
1,4-*cis* (%)	1,4-*trans* (%)		
15	56.4		

**Table 5:** Insoluble (gel) and soluble fractions of the SBR latex synthesized through seeded miniemulsion polymerization using in-situ generated surfactant and HD as the hydrophobe.

Entry	Insoluble Fraction (%)	Soluble Fraction (%)
**HD-SBR#01**	5.3	94.7
**HD-SBR#02**	5.8	94.2
**HD-SBR#03**	5.3	94.7
**Average**	**5.5**	**94.5**

**Table 6:** Properties of SBR latexes synthesized using HD and octadecyl acrylate (ODA) as the hydrophobe.

Sample	*T*_g_ (°C)	Molar Mass			Gel Content (%)	Composition (mol %)			
		*M*_w_ (g mol^−1^)	*M*_n_ (g mol^−1^)	Ð		Styrene	1.2-Vinyl	1.4-*cis*	1.4-*trans*
HD-SBR#01	−52	30,382	8040	3.8	5.3	11.9	15.7	14.5	57.9
HD-SBR#02	−56	31,664	6980	4.5	5.8	12.8	15.8	15	56.4
HD-SBR#03	−56	27,458	6177	4.4	5.3	13.0	16.2	15.2	55.6
ODA-SBR#01	−50	45,877	10,164	4.5	4.6	13.5	15.0	15.3	56.2
ODA-SBR#02	−50	47,992	10,369	4.6	3.2	13.2	15.5	15.5	55.8
ODA-SBR#03	−53	46,514	8288	5.2	3.8	12.9	14.9	15.3	56.9

## Supplementary Materials

The following are available online at https://www.mdpi.com/2073-4360/12/7/1476/s1, Figure S1: DSC analysis of the SBR latex obtained by seeded miniemulsion polymerization using in-situ generated surfactant and HD as the hydrophobe (HD-SBR#01), Figure S2: Effect of the t-DM content (wt% based on total monomer) on the molar mass and molar mass distribution of the HD-SBR latexes, Figure S3: DLS analysis of HD-SBR#01. (a) styrene miniemulsion droplets before polymerization and (b) final SBR particle diameter, Figure S4: DLS analysis of ODA-SBR#01. (a) styrene miniemulsion droplet before polymerization and (b) final SBR particle diameter, Figure S5: Droplet diameter, particle diameter and main characteristics of the HD-SBR latex obtained by scaling-up the recipe of Table 2 (total weight = 3 kg), Figure S6: Droplet diameter, particle diameter and main properties of up-scaled ODA-SBR latex (total weight = 3 kg), Figure S7. Cryo-TEM images of a SBR latex produced by miniemulsion polymerization using 2 wt% of VAZO 67, 3 wt% of in situ generated k-oleate and 4 wt% of t-DM as CTA, in the absence of co-stabilizer, Table S1: Insoluble (gel) and soluble fractions of the HD-SBR latex synthesized by seeded miniemulsion polymerization using 2 wt% of t-DM as CTA, Table S2: Molar masses of HD-SBR latex synthesized by seeded miniemulsion polymerization using 2, 3, and 4 wt% of t-DM as CTA, and Equations used to calculate the number of droplets (Nd) and the number of particles (Np).
