# Supplementary material for: Styrene-Butadiene Rubber by Miniemulsion Polymerization Using In Situ Generated Surfactant
Source: Polymers (Basel). 2020 Jun 30;12(7):1476. doi: 10.3390/polym12071476 (PMC7408609; doi:10.3390/polym12071476)
Supplement: Supplementary file 1 [file polymers-12-01476-s001.pdf]

polymers

Supplementary Materials

Styrene-Butadiene Rubber by Miniemulsion Polymerization using *in situ* Generated Surfactant

Anderson M. S. Medeiros[1], Elodie Bourgeat-Lami, and Timothy F.L. McKenna*

Univ Lyon, University Claude Bernard Lyon 1, CPE Lyon, CNRS, UMR 5265, Chemistry, Catalysis, Polymers and Processes (C2P2), 43 Bvd. Du 11 Nov. 1918, F-69616 Villeurbanne, France.

* Correspondence: Timothy.MCKENNA@univ-lyon1.fr (T.M.)

Equations used to calculate the number of droplets (N_d) and the number of particles (N_p)

$$N_p = \left(\frac{V_p^T}{V_{p_1}}\right)/M_{reactor}$$

where:

$$V_p^T = \frac{M_m}{\rho_m} + \frac{M_p}{\rho_p} + \frac{M_{Hydroph.}}{\rho_{Hydroph.}} = \frac{(1-X)M_m}{\rho_m} + \frac{X.M_m + M_{Initiator}}{\rho_p} + \frac{M_{Hydroph}}{\rho_{Hydroph}}$$

where: V_p^T = Total volume of all the particles; M_m = Mass of monomer; ρ_m= Density of monomer; M_p = Mass of polymer; ρ_p = Density of polymer; $M_{Hydroph.}$ = Mass of hydrophobe; $\rho_{Hydroph.}$= Density of hydrophobe; $M_{reactor}$ = Total mass

$$V_{p_1} = \frac{\pi}{6} D_p^3$$

where: V_{p1} = Volume of a single particle; D_p = Diameter of particles.

For N_d calculations, M_p/ρ_p was considered as zero because there are just nanodroplets at t=0.

[1] Current address : Laboratoire de Chimie des Polymères Organiques (LCPO, UMR 5629), ENSCBP/CNRS – Université de Bordeaux, 16 Avenue Pey Berland – 33607 Pessac, France.

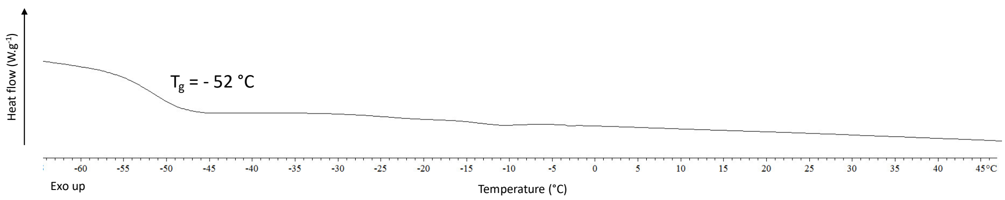

Figure S1. DSC analysis of the SBR latex obtained by seeded miniemulsion polymerization using in-*situ* generated surfactant and HD as hydrophobe (HD-SBR#01)

Table S1. Insoluble (gel) and soluble fractions of the HD-SBR latex synthesized by seeded miniemulsion polymerization using 2 wt. % of t-DM as CTA

Entry	Insoluble Fraction (%)	Soluble Fraction (%)
HD-SBR#01_2%CTA	22.7	77.3
HD-SBR#02_2%CTA	22.2	77.8
HD-SBR#03_2%CTA	22.1	77.9

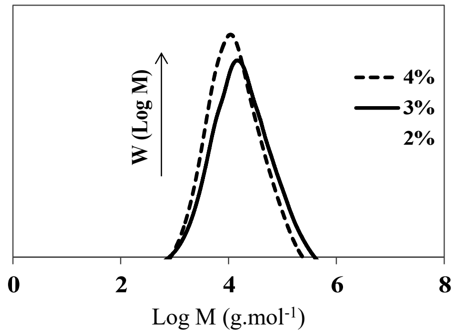

Figure S2. Effect of the t-DM content (wt% based on total monomer) on the molar mass and molar mass distribution of the HD-SBR latexes.

Table S2. Molar masses of HD-SBR latex synthesized by seeded miniemulsion polymerization using 2, 3, and 4 wt. % of t-DM as CTA

**t-DM * content (%)**	M_w **(g.mol^{-1})**	M_n**(g.mol^{-1})**	**Đ**
2	84 313	11 955	7.0
3	45 195	8 403	5.4
4	28 280	6 902	4.1

* tert-dodecylmercaptan

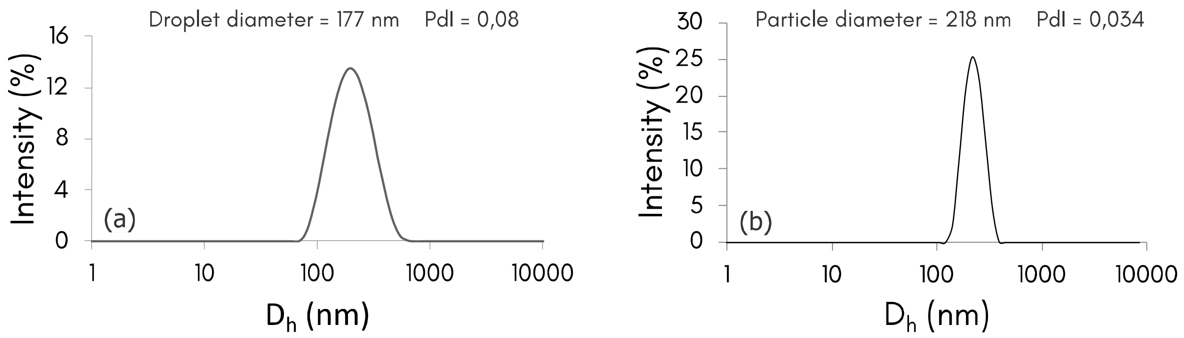

Figure S3. DLS analysis of HD-SBR#01. (a) styrene miniemulsion droplets before polymerization and (b) final SBR particle diameter.

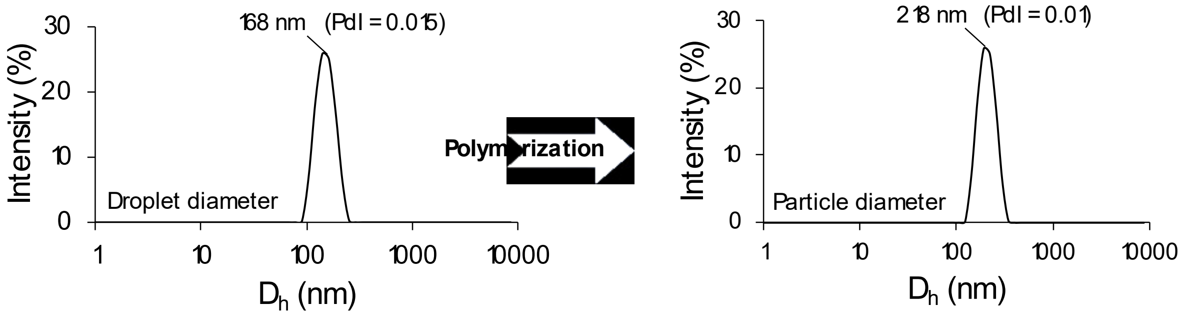

Figure S4. DLS analysis of ODA-SBR#01. (a) styrene miniemulsion droplet before polymerization and (b) final SBR particle diameter.

Hexadecane-based SBR Properties

Entry	Droplet Diameter (nm)	Droplet PdI
**3Kg-HD-SBR#01**	210	0.05
**3Kg-HD-SBR#02**	194	0.03
**3Kg-HD-SBR#03**	217	0.01

Polymerization →

Entry	Particle Diameter (nm)	Particle PdI
**3Kg-HD-SBR#01**	242	0.05
**3Kg-HD-SBR#02**	255	0.02
**3Kg-HD-SBR#03**	260	0.06

Solids content: 45%

Sample	Tg (°C)	Molar mass			Gel content (%)	Composition (%)			
		M_w (g mol^{-1})	M_n (g mol^{-1})	PdI		Styrene	1.2-vinyl	1.4-*cis*	1.4-*trans*
**3Kg-HD-SBR#01**	- 55	32112	9028	3.5	5.6	11.0	14.8	15.7	58.5
**3Kg-HD-SBR#01**	- 53	33324	9170	3.6	6.4	11.8	15.3	15.8	57.1
**3Kg-HD-SBR#01**	- 54	33119	8889	3.7	6.1	12.2	15.3	15.3	57.2

Figure S5. Droplet diameter, particle diameter and main characteristics of the HD-SBR latex obtained by scaling-up the recipe of Table 2 (total weight = 3 kg).

Octadecyl acrylate-based SBR Properties

Run	Droplet	
	Diameter (nm)	PdI
3Kg-ODA-SBR#01	198	0.02
3Kg-ODA-SBR#02	221	0.1
3Kg-ODA-SBR#03	237	0.1

Polymerization →

Run	Particle	
	Diameter (nm)	PdI
3Kg-ODA-SBR#01	217	0.02
3Kg-ODA-SBR#02	215	0.01
3Kg-ODA-SBR#03	230	0.02

Solids content: 45%

Sample	Tg (°C)	Molar mass			Gel content (%)	Composition (%)			
		M_w (g mol^{-1})	M_n (g mol^{-1})	PdI		Styrene	1.2-vinyl	1.4-*cis*	1.4-*trans*
**3Kg-ODA-SBR#01**	- 53	44973	9981	4.5	4.8	12.4	14.8	15.6	57.0
**3Kg-ODA-SBR#02**	- 51	46451	9222	5.0	4.8	12.3	14.4	15.4	57.9
**3Kg-ODA-SBR#03**	- 53	45411	10134	4.5	4.2	12.1	14.6	15.2	58.1

Figure S6. Droplet diameter, particle diameter and main properties of up-scaled ODA-SBR latex (total weight = 3 kg).

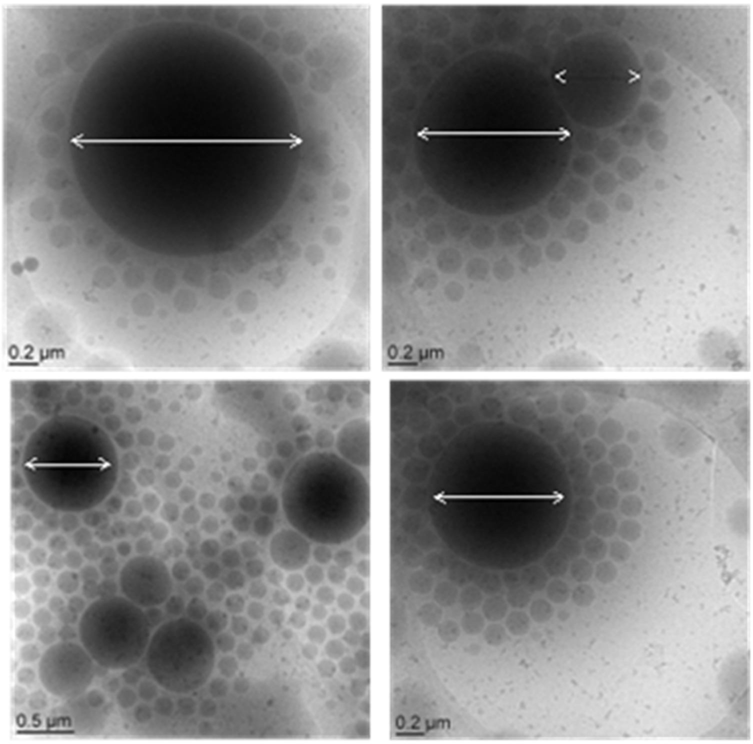

Figure S7. *Cryo*-TEM images of a SBR latex produced by miniemulsion polymerization using 2 wt. % of VAZO 67, 3 wt. % of *in situ* generated k-oleate and 4 wt. % of t-DM as CTA, in the absence of co-stabilizer.
